# Mechanisms by Which Plant Extracts Ameliorate Bovine Mastitis Through the Regulation of Mitochondrial Function: A Review

**DOI:** 10.3390/cells15131222

**Published:** 2026-07-06

**Authors:** Zhuojia Li, Jie Song, Changjin Ao, Lifang Wang, Tenglong Zhang, Yabo Zhao, Chenyang Guo, Huachen Zhong, Jialin Liu

**Affiliations:** 1 Key Laboratory of Animal Nutrition and Feed Science, College of Animal Science, Inner Mongolia Agricultural University, Hohhot 010018, China; lzjlzj0505@163.com; 2 Inner Mongolia Academy of Agricultural and Animal Husbandry Sciences, Hohhot 010031, China; songjiesau@163.com (J.S.);

**Keywords:** mastitis, mitochondrial dysfunction, mitochondrial repair, mitochondrial damage, plant extracts, targeted regulatory mechanism

## Abstract

**Highlights:**

**What are the main findings?**

- Disruption of the balance between mitochondrial repair and damage is among the key causes of mammary cell injury and apoptosis during bovine mastitis.
- Plant extracts alleviate bovine mastitis through dual mechanisms: promoting mitochondrial repair and limiting mitochondrial damage.

**What are the implications of the main findings?**

- This review provides a theoretical basis for using plant extracts to alleviate bovine mastitis through targeting mitochondrial function.
- It offers new approaches to antibiotic substitution for the green control of bovine mastitis.

**Abstract:**

Mastitis is recognized worldwide as one of the most expensive and common bovine diseases, severely affecting bovine health and milk quality. Mitochondria, known to play important roles in bovine mammary epithelial cells (BMECs), not only provide energy for milk synthesis but also participate in the regulation of the intracellular redox balance, inflammatory reactions, calcium signal transduction, and apoptosis. Mastitis destroys the dynamic balance between the intrinsic repair and damage mechanisms of mitochondria, which leads to mitochondrial dysfunction and aggravation of mammary cell injury or apoptosis. Plant extracts are rich in bioactive substances and are promising antibiotic substitutes for alleviating bovine mastitis. This paper reviews the mechanism through which plant extracts promote mitochondrial repair by interfering with mitophagy, dynamic balance and biogenesis, and alleviate mitochondrial damage by inhibiting mitochondrial ROS, calcium homeostasis imbalance and permeability changes, resulting in the regulation of the mitochondrial function of mammary cells under inflammation and oxidative stress. Elucidation of these mechanisms can provide new strategies for the targeted control of bovine mastitis.

## 1. Introduction

Mastitis is an inflammatory reaction that occurs in the bovine mammary gland owing to the invasion of pathogenic microorganisms or stimulation via physical and chemical factors, not only leading to reductions in milk yield and milk quality but also increasing the culling rate and breeding costs of dairy cows. Specifically, the economic losses caused by mastitis are as high as $35 billion worldwide every year [1]. Mastitis can be divided into recessive and clinical mastitis, with the former occurring 5–10 times as often as the latter [2]. In eukaryotic cells, mitochondria are important organelles; their double-layered structure contains mainly an outer mitochondrial membrane (OMM), an inner mitochondrial membrane (IMM), an intermembrane space (IMS) and a matrix [3]. In the mammary gland, the core function of mitochondria is the conversion of nutrients into direct energy through the production of ATP via oxidative phosphorylation, which provides energy for various physiological activities, such as proliferation and lactation, in bovine mammary epithelial cells (BMECs) [4,5]. During mastitis, under conditions of inflammation and oxidative stress, mitochondria exhibit increased reactive oxygen species (ROS) production [6], an imbalance in calcium homeostasis [7], membrane permeability changes [8] and other damage-related phenomena, thus affecting the structure and function of BMECs. A series of mitochondrial repair mechanisms, such as mitophagy [9], mitochondrial dynamics [10], and mitochondrial biogenesis [11], can eliminate damaged mitochondria and maintain mitochondrial balance to a certain extent, preventing excessive oxidative stress and inflammation from destroying mitochondria and cells; however, this protective function is inhibited when mitochondrial damage exceeds the repair capacity.

Currently, antibiotics are the first treatment of choice for bovine mastitis, but long-term use can lead to food safety risks such as pathogen resistance and antibiotic residues in milk [12]. Plant extracts are rich in polyphenols, flavonoids, tannins, alkaloids, essential oils and other bioactive components [13], and they exhibit antibacterial [14], antioxidant [15], and anti-inflammatory effects [16], among others, making them potential substitutes for antibiotics. In recent years, research on plant extracts has gradually shifted to the precise regulation of mitochondrial function, such as autophagy promotion, dynamic balance, mitochondrial ROS regulation, and calcium buffering, thus revealing their promising potential in the protection of mammary cells and the treatment of mastitis. Therefore, this paper seeks to review the mitochondria-targeting regulatory mechanisms of plant extracts in the prevention and control of mastitis from the perspectives of promoting mitochondrial repair and inhibiting mitochondrial damage, providing a theoretical basis for the development of green treatment strategies for mastitis based on natural products.

## 2. Methodology

This review was conducted as a narrative synthesis of the literature. The literature search was performed across multiple electronic databases, including PubMed, Web of Science, and Google Scholar. The search strategy was developed around the central topic, using combinations of the following terms: “bovine mastitis”, “mammary epithelial cells”, “plant extracts”, “mitochondrial dysfunction”, “mitochondrial damage”, “mitochondrial repair”, “mitochondrial targeted regulation mechanism”, “mitophagy”, “mitochondrial dynamics”, “mitochondrial biogenesis”, “mitochondrial reactive oxygen species”, “mitochondrial calcium homeostasis”, and “mitochondrial permeability”. The search covered manuscripts published from 2016 to 2026. The inclusion criteria were as follows: (1) studies directly relevant to the theme of the review; and (2) original research articles or reviews based on in vitro models (e.g., cells), in vivo rodent models, ruminant models, or dairy cow studies. The exclusion criteria included: (1) conference abstracts, short communications, or book chapters; (2) studies with incomplete data or notable flaws in experimental design; and (3) studies not aligned with the core topic of the review. Following the screening process, eligible publications were finally selected for comprehensive review and analysis.

## 3. Plant Extracts Promote Mitochondrial Repair

### 3.1. Plant Extracts Interfere with Mitophagy

**Figure 1:**
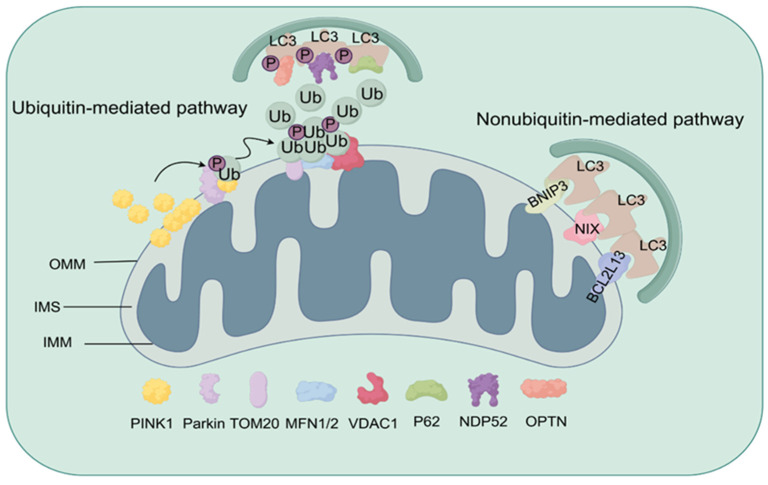
Two major mitophagy pathways. Ubiquitin-mediated mitophagy mainly involves the formation of Ub chains with proteins such as TOM20, MFN1/2 and VDAC1 on the OMM through the PINK1/Parkin pathway, which further promotes autophagy through autophagy factors such as OPTN, p62 and NDP52. Nonubiquitin-mediated mitophagy involves the binding of LC3 presented on autophagosomes to receptor proteins (BNIP3, NIX and BCL2L13) on mitochondria to promote autophagy.

Cells can selectively remove damaged or dysfunctional mitochondria via autophagy and deliver them to lysosomes for degradation to maintain stable mitochondrial quantity and quality [17]. Mitophagy plays pivotal roles in multiple physiological processes, including cellular inflammation, apoptosis [18], and senescence [19]. This process is regulated by two core pathways: the ubiquitin-mediated pathway and the nonubiquitin-mediated pathway (Figure 1). In mammals, the ubiquitin-mediated pathway is regulated primarily by the Ser/Thr kinase PINK1 and the E3 ubiquitin ligase Parkin. PINK1 is an important molecular sensor of mitochondrial health. Under physiological conditions, PINK1 is transported to the IMM via the translocase of the outer membrane (TOM)/translocase of the inner membrane (TIM) complex. PINK1 is subsequently cleaved by mitochondrial processing peptidase (MPP) and presenilin-associated rhomboid-like protease (PARL), resulting in its degradation [20,21]. When mitochondrial depolarization or protein misfolding is responsible for mitochondrial damage, PINK1 is unable to enter the IMM and accumulates in the OMM in large quantities. After dimerization and autophosphorylation occur, it becomes activated [22], triggering the phosphorylation of ubiquitin (Ub) and Parkin at the Ser65 site. These posttranslational modifications promote the recruitment of the cytoplasmic Parkin protein to damaged mitochondria [23], catalysing the ubiquitination of various proteins, such as the translocase of OMM 20 homologue (TOM20), mitofusin1/2 (MFN1/2), and voltage-dependent anion channel protein 1 (VDAC1), on the OMM through the formation of Ub chains [24], which are further phosphorylated by PINK1. Autophagy adaptor proteins such as optineurin (OPTN), sequestosome-1 (SQSTM1/p62), and nuclear dot protein 52 (NDP52) are activated in the mitochondria to recruit autophagy initiators such as unc-51-like kinase 1 (ULK1) and double FYVE domain-containing protein 1 (DFCP1) to promote mitophagy [25,26]. The nonubiquitin-mediated pathway is primarily activated following the expression of mammalian mitophagy receptors, such as BCL2/adenovirus E1B 19-kDa-interacting protein 3 (BNIP3), Nip3-like protein X (NIX), and B-cell lymphoma 2-like 13 (BCL2L13). These receptors all contain LIR structural domains that interact with microtubule-associated protein 1 light chain 3 (LC3), which binds to LC3 on the autophagy membrane, thereby targeting the autophagy machinery to the mitochondria. This process occurs independently of the ubiquitination pathway [18].

Mitophagy preserves mammary cell homeostasis by mitigating excessive oxidative stress and inflammatory responses [9,27]. The increased autophagic activity in the mammary glands of cows with mastitis may be attributed to their infection by pathogenic microorganisms that trigger mitophagy and clear ROS to alleviate the inflammatory reactions [28,29]. In a bovine mammary epithelial cell line (MAC-T), *Staphylococcus aureus* (*S. aureus*) invasion resulted in a reduction in the mitochondrial membrane potential (MMP) and significantly increased the protein expression levels of PINK1, Parkin, and LC3-II, triggering mitophagy. Silencing PINK1 inhibited mitophagy and aggravated mitochondrial ROS (mtROS) production, leading to the activation of the NLR family pyrin domain-containing protein 3 (NLRP3) inflammasome and the increased phosphorylation of the p65 subunit of nuclear factor kappa-B (NF-κB) and its inhibitor, inhibitor kappa B alpha (IκBα). These findings demonstrate that PINK1/Parkin-mediated mitophagy attenuates the inflammatory response triggered by *S. aureus* infection [27]. Similarly, Zhou et al. [30] reported comparable findings that *S. aureus* activates PINK1/Parkin-mediated mitophagy in bovine macrophages. In a lipopolysaccharide (LPS)-treated MAC-T cell inflammation model, PINK1-mediated mitophagy protected cells from inflammatory damage. PINK1 knockdown or cell pretreatment with the autophagy inhibitor 3-methyladenine inhibited the LPS-induced reduction in p62 protein expression, an increase in LC3-II protein expression, and greater numbers of autophagic lysosomes and autophagosomes. These changes ultimately resulted in increased production of mtROS and greater NLRP3, cleaved caspase-1, and interleukin-1β (IL-1β) protein levels. Conversely, PINK1 overexpression promoted mitophagy and attenuated LPS-triggered inflammatory responses [31]. However, another study demonstrated that *Escherichia coli* (*E. coli*) inhibited mitophagy in MAC-T cells and the mammary gland tissues of mice with mastitis, inducing ROS accumulation, NLRP3 inflammasome activation, and apoptosis [32]. These contradictory results may be attributable to differences in the pathogenicity of the pathogenic microorganisms or the duration of the infection. Additionally, metabolic stress, such as that observed in ketosis, also causes mammary gland damage in bovines and affects mitophagy. In a study involving both cows with ketosis and BMECs treated with free fatty acids (FFAs), low levels of FFAs activated autophagy and alleviated oxidative stress by increasing LC3-II and reducing p62 protein expression levels; conversely, oxidative stress induced by high levels of FFAs inhibited the autophagic process, leading to BMEC and mammary tissue injury in cows [33].

Plant extracts can be used as novel targeted regulators of mitophagy for preventing and treating mastitis. First, plant extracts target the PINK1/Parkin pathway to modulate mitophagy, thereby mitigating inflammation and oxidative damage. Forsythiaside A (FTA), a phenylethanoid glycoside isolated from *Forsythia suspensa*, has multiple pharmacological effects, including the induction of anti-inflammatory and anti-infection processes [34,35]. Liu et al. [5] reported that pretreatment with 15 µg/mL FTA for 12 h reduced LPS-induced mtROS production and the protein levels of the inflammatory factors TNF-α, IL-1β and IL-6 in MAC-T cells exposed to LPS while increasing the expression levels of the mitophagy-related proteins PINK1, Parkin and LC3. The mitophagy-inducing effects of FTA were attenuated upon treatment with the cyclosporine A (CsA) inhibitor or transfection with siPINK1. Similarly, the anti-inflammatory effects of the intragastric administration of 80 mg/kg FTA for 7 days significantly increased mitophagic activity in the mammary glands of mice with mastitis. Research on natural alkaloids has shown that treatment with 25 µM nitidine chloride for 2 h can reduce ROS production in BMECs under hypoxic conditions and reduce the expression levels of PINK1, Parkin, MFN1, VDAC1, and other related proteins in the PINK1–Parkin pathway. In this way, excessive mitophagy is inhibited, and oxidative stress and apoptosis are alleviated [36]. Second, plant extracts can activate AMP-activated protein kinase (AMPK), which is pivotal for the initiation of mitophagy. Research involving mastitis models has indicated that the AMPK/ULK1 axis plays a role in the plant extract-induced regulation of mitophagy [24,37]. Resveratrol is a characteristic polyphenolic compound that is abundant in plants such as peanut, grape, and mulberry [38]. This compound can activate mitophagy through AMPK activation and reduce the production of mtROS, IL-1β and NLRP3, thus exerting anti-inflammatory and antioxidant effects in various disease cell models [39]. FTA activates AMPK/ULK1 signalling to induce autophagy-associated LC3B protein expression and reduce p62 protein expression, mitigating apoptosis, oxidative stress, and inflammatory responses and thereby exerting a protective effect on MAC-T cells [40]. The lignan compound schisandrin A induces autophagy by activating the AMPK/ULK1 pathway after intraperitoneal injection at a concentration of 32 mg/kg in mice, thereby reducing the production of proinflammatory mediators and damage to mouse mammary epithelial cells induced by LPS stimulation [41]. In addition, studies on osteoarthritic injury, chronic kidney disease and other disease models have shown that AMPK can mediate the regulatory effects of plant extracts on AMPK/PINK1/Parkin-dependent mitophagy and nonubiquitin-mediated pathways, such as the AMPK/BNIP3/NIX pathway, thereby mitigating mitophagy [42,43]. Future research should explore the potential of plant extracts to ameliorate bovine mastitis through the regulation of the two mitophagy pathways in an AMPK-targeted manner.

### 3.2. Plant Extracts Maintain the Balance of Mitochondrial Dynamics

**Figure 2:**
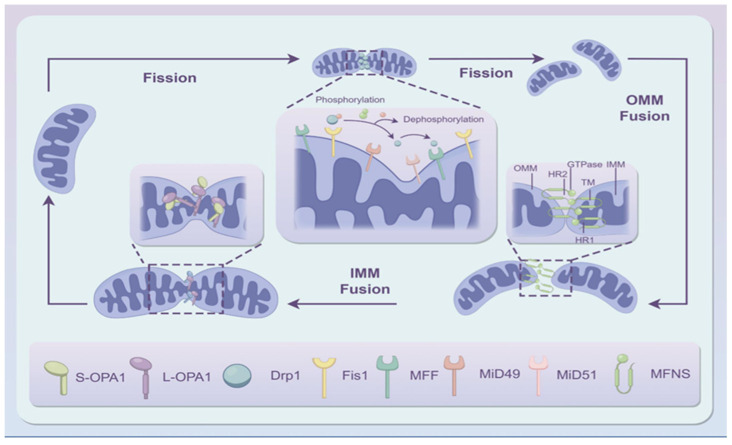
Dynamic mitochondrial processes. Mitochondrial dynamics involve two processes: fusion and fission. Mitochondrial fusion is mediated mainly by MFN1 and MFN2 in the OMM and the OPA1 protein in the IMM. Mitochondrial fission is mediated mainly by proteins such as Drp1, Fis1, MFF, MiD49 and MiD51.

Mitochondrial dynamics involve the processes of mitochondrial fusion and fission, which are essential for maintaining normal mitochondrial function and cellular homeostasis (Figure 2) [44]. Mitochondrial fusion is defined as the formation of a single mitochondrion from two or more mitochondria moving along microtubules. This process facilitates the mixing and exchange of mitochondrial constituents [45]. The mitochondrial fusion process can be divided into outer membrane fusion and inner membrane fusion. Outer membrane fusion is facilitated by MFN1 and MFN2, GTPases, which are localized on the OMM through two transmembrane (TM) domains. The N-termini contain a GTP-binding domain, a GTPase activity region and heptad repeat 1 (HR1), and the C-termini contain heptad repeat 2 (HR2). When multiple mitochondria approach each other, MFN1 and MFN2 interact in the cytosol to drive outer membrane fusion through GTP hydrolysis. The process of inner membrane fusion is mediated by optic atrophy 1 (OPA1), which resides in the IMM in two proteolytically processed forms: the long isoform (L-OPA1) and the short isoform (S-OPA1). Under basal conditions, OPA1 maintains the stability of the cristae architecture. During the fusion process, the GTPase function of OPA1 is induced, hydrolysing GTP to provide energy, thereby promoting IMM fusion [46].

Mitochondrial fission not only mediates mitochondrial intracellular movement and intracellular material partitioning during mitosis but also separates damaged mitochondrial regions for degradation via the mitophagy–lysosomal pathway [47]. Mitochondrial fission is mediated by dynamin-related protein 1 (Drp1); this GTPase is present mainly in the cytosol and is activated by the phosphorylation of Ser616 and the dephosphorylation of Ser637. Drp1 is subsequently recruited through OMM adaptor proteins such as fission 1 (Fis1), mitochondrial fission factor (MFF), mitochondrial dynamics protein of 49 kDa (MiD49) and mitochondrial dynamics protein of 51 kDa (MiD51) to a fission site that is tightened by the endoplasmic reticulum. Drp1 further drives its own oligomerization into a ring structure through GTP hydrolysis, promoting membrane contraction and mitochondrial division [45,48]. Among these proteins, Fis1 and MFF independently recruit Drp1, directly promoting mitochondrial division. While MiD49 and MiD51 also recruit Drp1, they maintain the protein in an inactive state until it is activated by cellular signalling [49].

Imbalanced mitochondrial dynamics are closely involved in mastitis. Heat stress (HS) is among the primary triggers of bovine mastitis, resulting in decreased milk yield and quality. Additionally, HS induces oxidative stress, which leads to increased ROS production and subsequent mitochondrial dysfunction [50]. Under HS conditions, BMECs exhibit increased expression of the mitochondrial fission proteins Drp1 and Fis1 and reduced expression of the fusion proteins MFN1 and MFN2. Typically, HS tends to promote mitochondrial fission to facilitate the removal of damaged mitochondria, thereby suppressing ROS accumulation and attenuating oxidative damage, whereas excessive fission may trigger mitochondrial dysfunction [10]. Nutritional metabolic diseases such as ketosis also induce mitochondrial dysfunction and oxidative stress in bovines [51], thereby increasing the incidence of mastitis [52]. Compared with that in healthy BMECs, the ROS content in a ketotic BMEC model increased, resulting in mitochondrial swelling and a reduced MMP, whereas the expression of the mitochondrial fusion-related protein MFN2 decreased significantly, indicating blockade of the normal functioning of mitochondria [53]. Furthermore, pathogenic microbial infections significantly affect mitochondrial homeostasis. Upon *E. coli* invasion of the BMECs and mammary glands of mice, the dynamic balance of the mitochondria is altered, and the fusion and fission processes are inhibited, eventually leading to inflammation; these effects are primarily attributed to a reduction in the mRNA expression levels of Drp1, Fis1, MFN1, MFN2 and OPA1 [54]. The *E. coli* virulence factor Map promoted mitochondrial fission by inducing the dephosphorylation of Drp1 at Ser637 and hindered mitochondrial fusion by reducing MFN1/MFN2 expression. These alterations triggered intrinsic apoptosis, characterized by increased caspase-3 p17 and Cytochrome c (Cyt c) levels, consequently disrupting MAC-T cell homeostasis and the health of the mammary glands of mice. Notably, treatment with the Drp1 inhibitor Mdivi-1 attenuated these pathological effects [55]. Zhou et al. [56] reported that *Streptococcus uberis* infection of mouse mammary epithelial cells resulted in the translocation of Drp1 to mitochondria. Moreover, they reported significant upregulation of Fis1 expression and downregulation of MFN-1 expression, leading to mitochondrial dynamics imbalance. AMPK, located upstream of Drp1, functions as its negative regulator. Both the AMPK activator AICAR and the Drp1 inhibitor Mdivi-1 can reverse the mitochondrial dynamics imbalance through AMPK/Drp1-dependent pathways, both of which can inhibit the increase in Drp1 phosphorylation levels and block Drp1 transport to the mitochondria.

Plant polyphenols, including flavonoids (such as flavones, flavanones, flavanols, flavonols, and anthocyanins) and nonflavonoid compounds (such as tannins, phenolic acids, and saponins), display significant advantages in regulating mitochondrial dynamics because of their potent antioxidant activity [57,58]. Previous studies have shown that plant extracts can promote mitochondrial fusion and prevent excessive mitochondrial fission. In a previous study, the addition of 25 μM natural polyphenolic compound procyanidin B2 for 24 h decreased the mRNA expression of the fission proteins Drp1 and Fis1 and significantly increased the mRNA expression of the fusion proteins MFN1 and OPA1. HS-induced dysfunction of mitochondrial dynamics in BMECs was subsequently reversed, and apoptosis and inflammatory damage were attenuated [59]. Another study indicated that BMECs treated with 25 µM dihydromyricetin, a flavonoid polyphenolic compound derived from *Ampelopsis grossedentata*, for 12 h attenuated mitochondrial dynamic dysregulation under HS conditions by maintaining the balance among mitochondrial fission and fusion-associated proteins. These effects primarily manifested as the downregulation of Fis1 and Drp1 mRNA expression and the upregulation of MFN1 and MFN2 mRNA expression [60]. In cows with mastitis, the circulating levels of IL-6 and IL-8 are significantly increased, whereas MFN1 and MFN2 mRNA expression is downregulated and Drp1 mRNA expression is upregulated, indicating an imbalance in mitochondrial dynamics during inflammation. Further verification in LPS-treated BMECs revealed that the addition of 15 µmol/L resveratrol, a nonflavonoid polyphenol compound, for 12 h significantly upregulated the expression of MFN1 and MFN2 mRNA, downregulated the expression of Drp1 mRNA, and inhibited apoptosis induced by the B-cell lymphoma-2 (Bcl-2) protein family member Bcl-2-associated X protein (Bax) and caspase-3 in BMECs [61]. In addition to polyphenolic compounds, biologically active nucleotides exert similar regulatory effects on mitochondrial dynamics. Zeng et al. [10] reported that under HS conditions, nicotinamide mononucleotide reduced the expression of the mitochondrial fission protein Fis1 and the phosphorylation of Drp1 in BMECs, increased the expression of the mitochondrial fusion proteins MFN1 and MFN2, and reversed the depolarization of the MMP, indicating that this compound can alleviate defects in mitochondrial dynamics caused by HS.

### 3.3. Plant Extracts Modulate Mitochondrial Biogenesis

**Figure 3:**
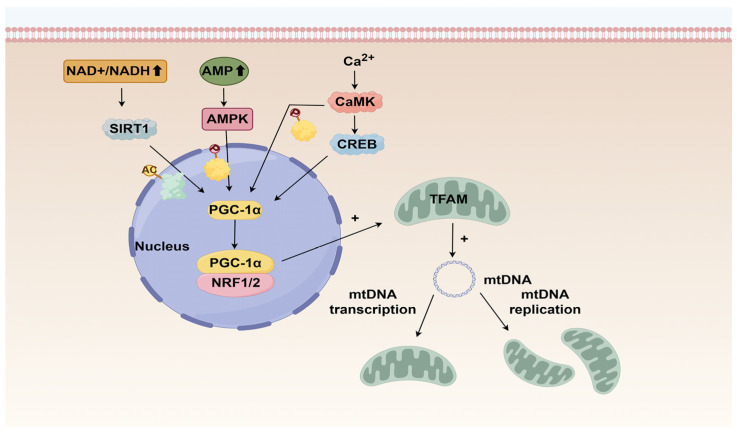
Regulation of mitochondrial biogenesis. Mitochondrial biogenesis is initiated mainly through the PGC-1α/NRF1/2/TFAM signalling pathway to promote mtDNA transcription and replication. PGC-1α is regulated by multiple upstream signalling pathways, such as the CaMK, AMPK and SIRT1 pathways.

Mitochondrial biogenesis refers to the process by which new mitochondria are produced in cells to meet increased energy and metabolic needs, including the synthesis and assembly of mitochondrial components such as proteins, lipids and DNA [62]. Mitochondrial biogenesis is regulated by multiple factors (Figure 3). Peroxisome proliferator-activated receptor gamma coactivator 1α (PGC-1α) is a key transcriptional regulator of mitochondrial biogenesis that initiates this process primarily through the PGC-1α/nuclear respiratory factor 1/2 (NRF1/2)/mitochondrial transcription factor A (TFAM) signalling pathway. In this nucleus, PGC-1α in the nucleus activates NRF-1 and NRF-2 expression through phosphorylation or deacetylation [63]. NRF-1 and NRF-2 further bind to specific sites to stimulate the TFAM promoter, induce TFAM expression, promote mitochondrial DNA (mtDNA) transcription and replication, and increase mitochondrial biogenesis. Activation of the PGC-1α-NRF-1/2 pathway can also upregulate the expression of the mitochondrial transcription factors B1 and B2 (TFB1M/TFB2M) and increase mtDNA transcription [64]. PGC-1α activation is regulated by various upstream signals, such as energy stress signals (AMPK pathway), calcium signals (calmodulin-dependent protein kinase (CaMK) pathway) and nutrient and metabolic signals (silent regulatory protein 1 (SIRT1) pathway). AMPK, a core factor involved in energy perception, plays an important role in regulating mitochondrial biogenesis. High levels of AMP result in AMPK activation, PGC-1α phosphorylation, and mitochondrial biogenesis-related gene expression. CaMK is another regulatory factor that drives mitochondrial biosynthesis; changes in the Ca^2+^ concentration activate CaMK, resulting in the phosphorylation of cyclic adenosine monophosphate response element binding protein (CREB), the induction of PGC-1α mRNA expression, or the direct phosphorylation of the PGC-1α protein to increase its transcriptional activity by inducing a conformational change [65]. In addition, the degree of nutritional metabolism affects the ratio of oxidized nicotinamide adenine dinucleotide (NAD^+^) to reduced nicotinamide adenine dinucleotide (NADH). At high ratios, activated SIRT1 deacetylates PGC-1α, relieves its self-inhibiting conformation, and enhances its binding to the NRF1/2 transcription factors [66,67].

Multiple studies have shown that mammary inflammation and oxidative stress can cause abnormal mitochondrial biogenesis. *E. coli* infection or treatment with the virulence factor LPS significantly downregulated the expression of PGC1-α, NRF1, TFAM and mitochondrial DNA displacement loop (D-Loop) mRNA in MAC-T cells and mouse mammary glands; mitigated mitochondrial biogenesis; decreased mitochondrial quality; and triggered inflammatory responses in BMECs and mouse mammary glands [54]. A high-concentration diet reduced ruminal pH and increased the LPS content in the blood of cows; subsequently, the LPS migrated to the mammary gland, inducing acute mastitis, which significantly activated the mitogen-activated protein kinase (MAPK) inflammatory signalling pathway in the mammary gland; increased the protein expression of the inflammatory markers IL-6, TNF-α, IL-1β and myeloperoxidase (MPO); and decreased the protein levels of SIRT1, PGC-1α, NRF1 and TFAM, ultimately blocking mitochondrial biogenesis [8]. Ketosis is a common metabolic condition in high-yielding cows. When cows have a negative energy balance, the levels of nonesterified fatty acids (NEFAs) increase, which in turn leads to elevated levels of the main components of ketone bodies, such as β-hydroxybutyrate (BHB), acetoacetate and acetone. Through in vivo experiments, Li et al. [68] reported that in ketotic cows, the serum levels of NEFAs and BHB were significantly elevated, while PGC-1α protein expression in mammary tissue was markedly downregulated. These changes were accompanied by mitochondrial dysfunction, reduced ATP content, and impaired mammary gland function, ultimately leading to a significant decrease in milk yield. In vitro mechanistic studies further demonstrated that treating MAC-T cells with NEFAs similarly suppressed PGC-1α protein expression, reduced ATP levels and the MMP, and induced mitochondrial dysfunction and oxidative stress. In addition, BMEC treatment with high levels of acetoacetic acid also led to significantly increased ROS levels; an attenuated MMP; decreased expression of PGC-1α, TFAM, and NRF1 mRNA; reduced mtDNA and ATP contents; and disruption of the steady state of mitochondrial biogenesis [69].

Regulation of the PGC-1α/NRF-1/TFAM signalling axis by plant extracts is the most important way to promote mitochondrial biogenesis in BMECs. Hu et al. [61] reported that PGC-1α activation was blocked in the blood of cows with mastitis. Mechanistically, the addition of 15 μmol/L resveratrol, a representative polyphenol, for 12 h acts as a PGC-1α agonist, significantly increasing the expression of PGC-1α and downstream TFAM mRNA in LPS-treated BMECs and promoting mitochondrial biogenesis. AMPK catalytic subunit α1 (PRKAA1) plays a key role in this process; after si-PKAA1 transfection, the positive effects of resveratrol on the regulation of mitochondrial biogenesis and the protection of BMECs from inflammatory damage through PGC-1α were weakened. Similar results have been reported by Ma et al. [70]; after an anti-PGC-1α intervention, the addition of 15 µmol/L resveratrol for 24 h did not inhibit the upregulation of the mRNA expression of inflammatory factors IL-6 and IL-8, oxidative factor malondialdehyde (MDA) or apoptotic factor Bax in LPS-treated BMECs, indicating that the sensitivity of the BMECs to inflammation and oxidative stress was reduced after mitochondrial biogenesis inhibition. Sulforaphane is an antioxidant isothiocyanate present in cruciferous plants such as broccoli and cabbage. Adding 10 µmol/L sulforaphane for 24 h activated nuclear factor erythroid 2-related factor 2 (Nrf2), alleviated the acetoacetate-induced increase in the mtROS content and decrease in the MMP in BMECs, and promoted PGC-1α, TFAM and NRF1 mRNA expression and mtDNA synthesis associated with mitochondrial biogenesis [69]. In addition, the effects of saponins such as astragaloside IV, ginsenosides and total saponins from *Panax notoginseng* on BMECs have not been reported, but in cardiomyocytes, hepatocytes, endothelial cells and other cells, these compounds promote mitochondrial biosynthesis by regulating the PGC-1α/NRF1/TFAM signalling pathway [71]. Therefore, the development of other active components in plant extracts with similar effects or new targets to regulate mitochondrial biosynthesis is a potential strategy for ameliorating mastitis and oxidative damage.

## 4. Plant Extracts Inhibit Mitochondrial Damage

### 4.1. Plant Extracts Reduce mtROS Levels

**Figure 4:**
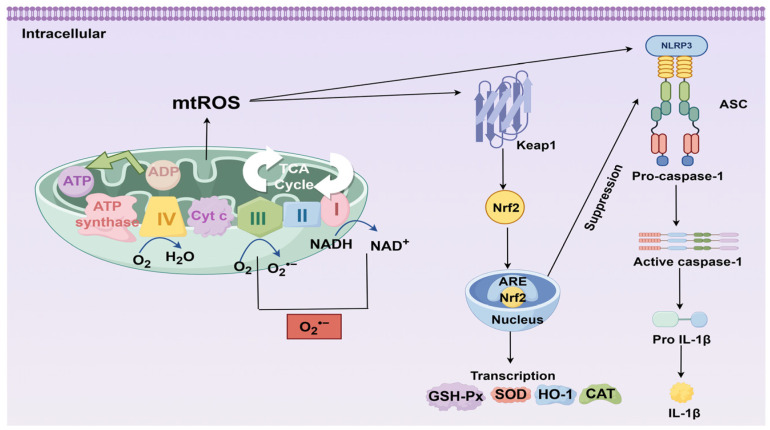
Generation of mtROS and mtROS signalling. Small numbers of electrons leak from the oxidative phosphorylation of the electron transport chain, generating O_2_^•−^ with oxygen molecules, which is then converted to mtROS such as H_2_O_2_ and ·OH, causing oxidative stress. These mtROS can activate the NLRP3 inflammasome, promote the release of inflammatory factors, and cause inflammation. They also promote the binding of Nrf2 and ARE and maintain redox balance by regulating the transcription of antioxidant enzymes such as SOD, GSH-Px, CAT and HO-1.

Mitochondria, the organelles responsible for most oxygen consumption within cells, account for approximately 90% of cellular ROS [72]. mtROS are derived from the oxidative phosphorylation of the electron transport chain. In this process, approximately 0.2–2.0% of the electrons are not properly transferred to the final oxygen molecule to produce water [73]; instead, they leak primarily from Complexes I (NADH dehydrogenase) and III (ubiquinol-cytochrome c reductase). These leaked electrons react directly with oxygen molecules to generate superoxide anions (O_2_^•−^) (Figure 4), which further undergo conversion into hydrogen peroxide (H_2_O_2_) and the hydroxyl radical (·OH) [74]. Under physiological conditions, the generation and elimination of mtROS are balanced; disruptions to this balance may lead to the accumulation of mtROS, resulting in oxidative stress [72]. Oxidative stress is significantly associated with inflammatory responses, triggered by the ROS modulation of inflammatory pathways [75]. mtROS are recognized as critical triggers of NLRP3 inflammasome activation [76]. The NLRP3 inflammasome is a multiprotein complex primarily composed of NLRP3, apoptosis-associated speck-like protein (ASC), and caspase-1. Upon activation, the NLRP3 inflammasome oligomerizes to activate caspase-1, which facilitates the maturation and secretion of proinflammatory cytokines such as IL-1β, thereby triggering inflammatory responses and inducing pyroptosis [28]. In addition, mtROS can activate the key antioxidant defence transcription factor Nrf2 in response to oxidative stress, thereby mitigating the redox process [77]. Under normal circumstances, Kelch-like ECH-associated protein 1 (Keap1) functions by anchoring Nrf2 within the cytoplasm, thereby impeding its nuclear translocation [78]. After oxidative damage occurs, Nrf2 detaches from the Keap1 protein and is translocated to the nucleus, where it binds to the antioxidant response element (ARE), thereby regulating the transcription of various antioxidant enzymes, including superoxide dismutase (SOD), glutathione peroxidase (GSH-Px), catalase (CAT), and haem oxygenase-1 (HO-1) [79]. These antioxidant enzymes play crucial roles in scavenging mtROS, maintaining intracellular redox homeostasis, inhibiting the NLRP3 inflammasome and reducing inflammatory responses [80].

Oxidative stress, which is induced by conditions such as metabolic alterations and pathogenic microbial challenges, can lead to elevations in ROS levels, mitochondrial dysfunction, and impaired immune activity, thus significantly increasing the risk of mastitis in periparturient cows [81,82]. The levels of oxidative stress markers were significantly increased in the milk of cows with *S. aureus*-induced subclinical mastitis [83]. Compared with healthy mammary tissue, bovine mammary glands with mastitis exhibit impaired mitochondrial structure and function, as well as elevated levels of ROS and the inflammatory mediators IL-1β, IL-6, TNF-α, MPO, and inducible nitric oxide synthase (iNOS) [6]. Mechanistically, the Keap1/Nrf2 system, a crucial element of antioxidant defence in mammary epithelial cells, plays a core protective role in counteracting ROS-induced damage and preventing mastitis [84]. Mastitis caused by infection with a pathogenic microorganism can induce oxidative stress and inflammation by disrupting the Keap1/Nrf2 antioxidant axis. Specifically, *Prototheca bovis* (*P. bovis*) significantly suppressed the expression of Nrf2, Keap1, and SOD proteins in the mammary gland tissues of mice and in mammary epithelial cells but increased the levels of mtROS, MDA, and HO-1, leading to mitochondrial structural damage and oxidative injury in mammary tissue. Scavenging mtROS or activating Nrf2 suppresses the expression of proteins in the NF-κB/NLRP3 inflammasome pathway, thereby alleviating *P. bovis*-induced oxidative stress and inflammatory responses [85]. Further validation in BMECs revealed that *P. bovis* stimulation resulted in the accumulation of mtROS, activation of the NF-κB/NLRP3 inflammatory signalling pathway, and increased levels of pro-caspase1, caspase1 p20, pro-IL-1β, and IL-1β proteins, ultimately triggering inflammatory responses and mitochondrial damage [82]. Similarly, *Klebsiella pneumoniae* (*K. pneumoniae*) infection increases ROS and MDA levels; reduces SOD activity, CAT activity and total antioxidant capacity (T-AOC); reduces the protein expression of HO-1 and NAD(P) H: quinone oxidoreductase 1 (NQO1) in the Nrf2/Keap-1 signalling pathway; and disrupts the antioxidant capacity of BMECs. In addition, through activation of the NF-κB pathway via the phosphorylation of the IκBα and p65 proteins, *K. pneumoniae* induces inflammatory responses, leading to cell damage [79].

Plant extracts can regulate free radical levels and oxidative stress-related Nrf2/ARE activity and reduce the accumulation of mitochondrial and intracellular ROS, thereby alleviating oxidative damage and inflammation in mammary tissue and cells. Many studies have shown that polyphenolic compounds, which are among the most effective antioxidants and anti-inflammatory compounds in plants, have promising potential for protecting mammary cells. The addition of 10 µM curcumin for 24 h activated the Nrf2-ARE pathway in LPS-injured BMECs; subsequently increased the levels of HO-1, NQO1, T-AOC, T-SOD and GSH; attenuated the production of ROS and MDA; and restored the MMP to normal values. Moreover, curcumin prevented the activation of the NF-κB and caspase/Bcl-2 pathways and alleviated oxidative stress and inflammation [86]. Adding 120 µg/mL hesperidin for 24 h effectively attenuated H_2_O_2_-induced damage to MAC-T cells, increased MMP stability and activated the Keap1/Nrf2/ARE signalling pathways by inducing Nrf2 nuclear translocation. These processes upregulated downstream NQO1 and HO-1 mRNA and protein expression, increasing antioxidant enzyme activity and scavenging ROS [87]. Hydroxytyrosol, which is present in the fruits and leaves of *Olea europaea* L., was added at concentrations of 10 µM and 25 µM for 1 h to exert protective anti-inflammatory effects by suppressing the levels of oxidative stress markers, including ROS, NADPH oxidase-1 (NOX-1) and proinflammatory cytokines (TNF-α, IL-1β, and IL-6), in LPS-injured MAC-T cells. This mechanism is associated with the activation of the Nrf2 signalling pathway and the upregulation of the mRNA expression levels of downstream antioxidant enzymes, including HO-1, NQO-1, and thioredoxin reductase 1 (Txnrd1) [88]. *Taraxacum officinale* extract, which is rich in flavonoids and phenolic acids, can specifically activate the Nrf2/ARE pathway, promote the transcription of downstream antioxidant genes such as HO-1 and NQO1, and inhibit ROS production, thereby significantly attenuating LPS-induced MAC-T cells oxidative stress; the optimal addition amount was 50 µg/mL and 48 h, respectively [89].

Furthermore, the in vivo and in vitro antioxidant and anti-inflammatory activities of polysaccharides have been substantiated through extensive research. For example, lentinan performs various biological functions. First, lentinan exerts free radical scavenging effects through the specific chelation of ferrous iron ions, blocking the Fenton reaction and mitigating the generation of the most destructive ROS, such as ·OH, at the source [90]. Second, treatment with 100 µg/mL lentinan for 6 h activated the endogenous antioxidant defence system through increased activation of the Nrf2 signalling pathway, thereby reducing the level of mtROS. Lentinan also alleviates LPS-induced damage to BMECs by inhibiting NF-κB and MAPK-related inflammatory pathways and preventing activation of the caspase-3-regulated mitochondrial apoptosis pathway [91]. Administration of 50, 100, or 200 mg/kg *Astragalus* polysaccharide to lactating mice for 6 days yielded protective effects, including the prevention of mtROS overproduction, control of the ROS/NLRP3 signalling pathway and inhibition of inflammatory factor expression. Furthermore, it restored the redox balance in the mammary glands of mice and MAC-T cells exposed to LPS, thereby preventing inflammation [92].

### 4.2. Plant Extracts Prevent the Mitochondrial Calcium Homeostasis Imbalance

**Figure 5:**
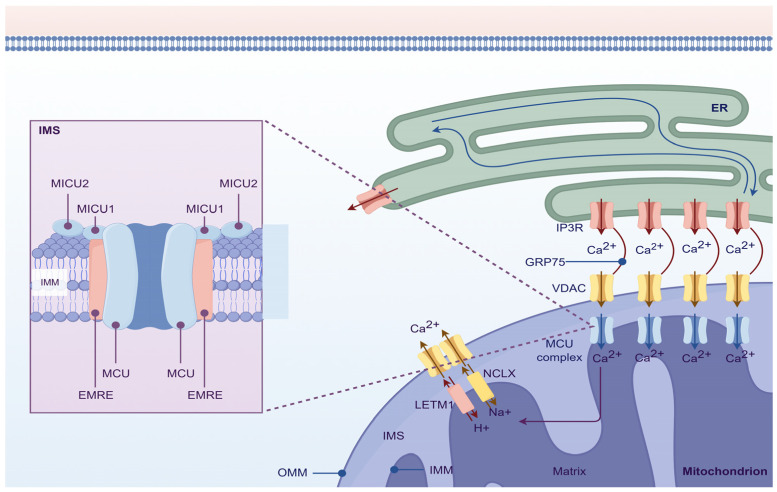
Process of mitochondrial Ca^2+^ transport. Mitochondria regulate Ca^2+^ metabolism through multiple coordinated channels, transporters and protein complexes to maintain calcium homeostasis. The OMM VDAC regulates Ca^2+^ permeability in a voltage-dependent manner, while the IMM MCU complex mediates the transport of mitochondrial matrix Ca^2+^. The IP3R-GRP75-VDAC-MCU signalling axis in MAMs mediates directional Ca^2+^ transport from the endoplasmic reticulum to the mitochondria; NCLX and LETM1 can also promote Ca^2+^ efflux and maintain mitochondrial Ca^2+^ homeostasis.

As intracellular Ca^2+^ regulators, mitochondria maintain calcium homeostasis through calcium ion uptake, storage, release and efflux, thus ensuring the orderly progression of mitochondrial respiratory function and ATP production (Figure 5) [93]. VDAC is the main calcium channel in the OMM that positively regulates mitochondrial calcium uptake. It consists of three subtypes: VDAC1, VDAC2 and VDAC3 [94]. This channel precisely regulates calcium ion permeability through voltage-dependent conformational changes: in the low-potential state, VDAC is open, and its ion selectivity is minimal; when the potential is high, the channel closes, increasing its ion selectivity [95]. The mitochondrial calcium uniporter (MCU) complex, located in the IMM, mediates the transmembrane transport of mitochondrial Ca^2+^. It is composed mainly of MCU, essential MCU regulator (EMRE), calcium uptake protein 1 (MICU1) and calcium uptake protein 2 (MICU2) [96]. Although the role of MCU regulatory protein 1 (MCUR1) as a component of the MCU complex remains controversial, its deletion disrupts oxidative phosphorylation, reduces the cellular ATP content, and inhibits mitochondrial Ca^2+^ uptake [97]. Mitochondrial-associated membranes (MAMs) function as dynamic connections between mitochondria and endoplasmic reticulum membrane contact points. Its core protein complex includes inositol 1,4,5-trisphosphate receptor (IP3R) on the endoplasmic reticulum and VDAC on the OMM, which jointly form a molecular bridge through glucose-regulated protein 75 (GRP75) [98]. This complex mediates the entry of endoplasmic reticulum Ca^2+^ into the IMS through VDAC and then the matrix through MCU in the IMM, thus constituting the IP3R–GRP75–VDAC–MCU calcium signalling axis and regulating mitochondrial Ca^2+^ homeostasis [94,99]. Mitochondrial Ca^2+^ efflux is mediated mainly by Na^+^/Ca^2+^/Li^+^ exchange protein (NCLX) and mitochondrial H^+^/Ca^2+^ exchange protein (LETM1) in the IMM [100]. Under physiological conditions, NCLX excretes mitochondrial Ca^2+^ in its positive mode (dependent on the Na^+^ gradient) to prevent overload; in pathological states, it shifts to a reverse mode, which mediates the internal flow of Ca^2+^ to aggravate damage [96]. LETM1 transports Ca^2+^ into the mitochondrial matrix and excretes H^+^ in the presence of low cytoplasmic levels of Ca^2+^. When the mitochondrial Ca^2+^ concentration is high, LETM1 and NCLX synergistically excrete Ca^2+^ to maintain the steady-state functioning of the mitochondria [101].

The mammary glands of lactating cows are the core sites of Ca^2+^ metabolic activity. During bovine mastitis, proinflammatory factors such as TNF-α and IL-1 disrupt calcium homeostasis, leading to increased cytoplasmic Ca^2+^ levels and thereby increasing the mitochondrial burden. Under conditions of persistent inflammation, mitochondrial Ca^2+^ overload may inhibit ATP synthesis and induce oxidative stress, further exacerbating cell damage. Therefore, by regulating energy metabolism and Ca^2+^ buffering, mitochondria play key roles in resistance to inflammatory damage in mammary tissue and in maintaining cellular homeostasis [7]. Research on the pathogenic microorganisms responsible for mastitis has revealed that *K. pneumoniae*, a main causative pathogen, can cause mitochondrial damage by disrupting Ca^2+^ homeostasis in BMECs. Six hours after infection with two strains (HB-AF5 and HLJ-D2) of *K. pneumoniae*, mitochondria exhibited a reduction in the MMP, excessive Ca^2+^ transfer, and overload of the Ca^2+^ level, which subsequently triggered pathological changes such as mitochondrial swelling, cristae structural alterations, vacuolization and even membrane rupture [102]. HLJ-D2 had a greater effect on the mitochondrial Ca^2+^ level than HB-AF5 did and induced significant increases in mitochondrial and cytoplasmic Ca^2+^ levels in BMECs [103]. Sun et al. [97] stimulated BMECs with LPS, which not only promoted MCUR1 transcription and translation but also increased the levels of mitochondrial Ca^2+^ and mtROS and reduced the MMP, resulting in mitochondrial damage and increased apoptosis. However, these negative effects were eliminated by interfering with MCUR1 gene function, indicating that MCUR1-mediated mitochondrial calcium transport is associated with mitochondrial damage during the inflammatory response in BMECs. In addition, mastitis-driven imbalance in Ca^2+^ signal transduction in the endoplasmic reticulum and mitochondria is closely related to changes in MAM function. Meng et al. [104] reported that treating BMECs with 2-APB (an IP3R inhibitor) reversed the increases in IP3R, GRP75, VDAC1 and MCU protein expression and the Ca^2+^ level induced by LPS. In contrast, Hu et al. [98] analysed the protein expression levels of MAM components in the mammary gland tissue of cows with mastitis and reported that the protein expression levels of IP3R, GRP75, and VDAC were significantly reduced. Meanwhile, in H_2_O_2_-treated BMECs, the connections between the endoplasmic reticulum and mitochondria were weakened, the abundances of key structural MAM molecules, such as MFN2 and IP3R, were reduced, and Ca^2+^ signalling was blocked.

The regulatory effects of plant extracts on mitochondrial calcium homeostasis closely involve the IP3R/GRP75/VDAC1/MCU axis. β-carotene is a natural carotenoid with antioxidant and anti-inflammatory effects, and treatment at 80 µM for 6 h effectively reduced LPS-induced inflammatory injury in BMECs. First, its protective mechanism involves inhibition of store-operated calcium entry (SOCE), which is composed of a cell membrane channel protein (calcium release-activated calcium channel protein 1, ORAI1) and an endoplasmic reticulum membrane protein (stromal interaction molecule 1, STIM1), reduction of intracellular calcium levels and blockade of NF-κB activation [105]. Second, β-carotene inhibits endoplasmic reticulum stress and activation of the MAM region through regulation of the STIM1-mediated IP3R/GRP75/VDAC1/MCU axis, thereby reducing Ca^2+^ levels. Treatment with 80 µM β-carotene for 12 h or intragastric administration of 100 mg/kg β-carotene for 14 days alleviated mitochondrial oxidative damage caused by excessive ROS production by BMECs and mouse mammary glands treated with LPS [104]. MFN2 stabilizes the MAM structure by promoting mitochondrial–endoplasmic reticulum fusion during cellular stress responses [106]. Studies on plant polysaccharides have shown that those from the seaweed *Gracilaria lemaneiformis* target MFN2, significantly alleviating bovine mastitis. The infusion of *G. lemaneiformis* polysaccharide (1 mg/mL) into the mammary glands of cows with mastitis twice daily for 10 d helped reduce the degrees of oxidative stress and inflammation and repaired damage to the function of the mammary gland. In this way, the polysaccharide not only reduced the serum levels of the inflammatory cytokines TNF-α, IL-6 and IL-1β and increased the levels of the antioxidant markers SOD and GSH-Px but also significantly reduced the somatic cell count (SCC) of milk and increased milk yield. The underlying mechanism involves increased MFN2 protein levels in oxidatively damaged MAC-T cells after the addition of 100 µg/mL *G. lemaneiformis* polysaccharide for 12 h, which effectively restored MAM integrity and maintained cellular calcium homeostasis [98]. Studies on alkaloids have shown that treatment with ryanodine, derived mainly from the stems of tropical *Salicaceae* plants [107], at 10 µmol/L for 30 min weakens mitochondrial Ca^2+^ overload and reduces the levels of excess mtROS in MAC-T cells challenged with LPS, similar to the effect of MCUR1 knockdown [97].

### 4.3. Plant Extracts Prevent Changes in Mitochondrial Permeability

**Figure 6:**
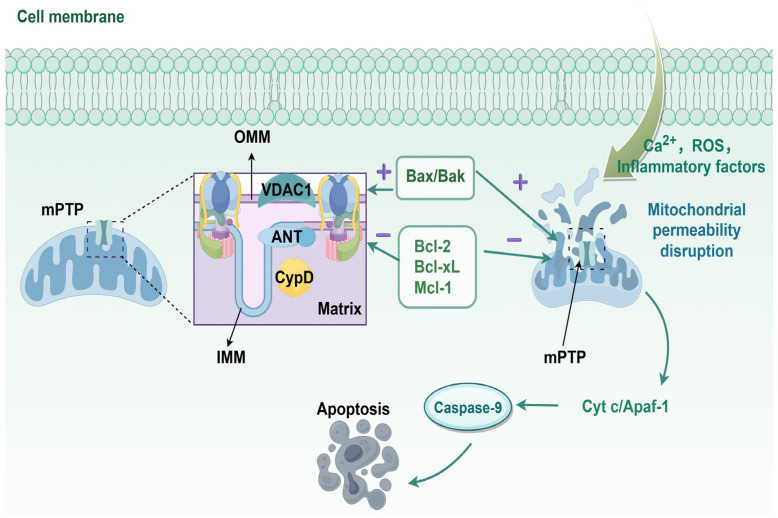
Mechanism of mitochondrial permeability regulation. Mitochondrial permeability closely involves members of the Bcl-2 protein family and the mPTP. Activated Bax/Bak is inserted into the OMM and forms oligomers, which increase the permeability of the OMM. Then, Cyt c is released and forms apoptotic bodies with Apaf-1, which activates the caspase-9 apoptosis pathway. Opening of the mPTP (involving VDAC1, ANT, and CypD) increases the permeability of the IMM and decreases the MMP. This opening is regulated by the levels of Ca^2+^, ROS and inflammatory factors.

Changes in mitochondrial permeability occur mainly between the OMM and IMM, which are the core components that regulate apoptosis and cell necrosis processes (Figure 6) [108]. Studies have shown that the Bcl-2 protein family is closely involved in this process. The Bcl-2 family of proteins can be divided into antiapoptotic proteins such as Bcl-2, myeloid cell leukaemia 1 (Mcl-1), and B-cell lymphoma-xL (Bcl-xL) and proapoptotic proteins such as Bcl-2-interacting mediator of cell death (Bim), BH3-interacting domain death agonist (Bid), Bcl-2 homologous antagonist (Bak), and Bax. The C-termini of these proteins share a hydrophobic region that anchors the organelle membrane, while their peptide chains contain four conserved BH1–BH4 helix domains. Among these proteins, the proapoptotic proteins Bax/Bak, which contain all BH domains, directly participate in the apoptosis program, whereas the Bim and Bid proteins, which contain only BH3 domains (BH3-only proteins), regulate cell survival by binding to the hydrophobic grooves on the surface of antiapoptotic proteins and forming heterodimers [109]. When the cell receives the apoptotic signal transmitted by the activated BH3-only protein, Bax/Bak are activated, insert into the OMM, and aggregate to form oligomers, which increases the permeability of the OMM. Cyt c is subsequently released into the cytoplasm and combines with apoptotic protease activating factor 1 (Apaf-1) to form apoptotic bodies, which then recruit and activate caspase-9 and initiate a caspase-3-dependent apoptosis cascade [9]. When cells necrose, changes in mitochondrial permeability transition pore (mPTP) permeability destroy the IMM and lead to continuous mitochondrial depolarization [108]. The mPTP, located in the IMS, is a multisubunit complex composed of a variety of proteins, chief among them VDAC1 in the OMM, adenine nucleotide translocase 1 (ANT1) in the IMM and cyclophilin D (CypD) in the matrix. Under normal circumstances, the mPTP is closed, preventing the release of proapoptotic factors such as Cyt c. After the pore opens, the permeability of the IMM increases sharply, and the MMP dissipates, leading to apoptosis or necrosis. mPTP opening is regulated by many factors, including the mitochondrial calcium level, ROS level, pH, and the levels of different inflammatory factors [110]. Studies have shown that antiapoptotic family members such as Bcl-2 and Bcl-xL inhibit mPTP opening, whereas proapoptotic family members such as Bax/Bak oligomerize to induce mPTP opening [109]. Additionally, under pathological conditions, CypD reduces the mPTP opening threshold by binding to ATP synthase, inducing mitochondrial damage and cellular necrosis [111].

Numerous studies have confirmed that changes in mitochondrial permeability are among the common pathways through which different pathogenic mechanisms trigger bovine mastitis, which may eventually lead to mammary tissue damage and mammary cell apoptosis [112]. Following maintenance on a high-concentrate diet, cows show symptoms of subacute ruminal acidosis, accompanied by increased LPS levels in the ruminal fluid and blood [113], which further significantly activate the mammary MAPK signalling pathway to increase the expression of IL-6, TNF-α and IL-1β and reduce the levels of ATP, resulting in mammary tissue damage. Moreover, high-concentrate diet-induced mastitis in cows upregulates the mRNA and protein expression of ANT, VDAC1 and CypD; promotes the opening of the mPTP; increases the permeability of the mitochondrial membrane; and elevates the level of Ca^2+^ in BMECs, thereby further destroying the integrity of mammary tissue [8]. Oxidative stress triggered by ketosis can simultaneously activate inflammatory response (NF-κB and NLRP3) and apoptosis (Bax/caspase-9/caspase-3) pathways, disrupt mitochondrial permeability and increase mastitis susceptibility in cows [114]. This process was also confirmed in an acetoacetate-induced ketosis BMEC model: excessive production of ROS reduced the MMP, indicating an imbalance in mitochondrial permeability [70]. In addition, a study on bovine mastitis caused by infection with pathogenic microorganisms also confirmed changes in mammary mitochondrial permeability. The expression levels of IL-1β, IL-6, TNF-α and ROS significantly increased, while MMP and VDAC1 protein expression significantly decreased in the mammary glands of cows with *E. coli*-induced mastitis, which resulted in the release of Cyt c [6]. The virulence factor Map from *E. coli* induces a reduction in the MMP in MAC-T cells, while the continuous opening of the mPTP leads to abnormal mitochondrial membrane permeability, which promotes the release of Cyt c and Ca^2+^ from the mitochondria to the cytoplasm, activates the Bax and caspase-3 proteins and inhibits Bcl-2 protein expression, thus inducing apoptosis [55]. Infection with the mastitis pathogen *P. zopfii* GT-II causes changes in mitochondrial permeability and in the MMP and triggers the classical apoptosis pathway mediated by Cyt c/Apaf-1, which is characterized by the formation of apoptotic bodies and activation of the caspase-9 and caspase-3 cascades [115].

Plant-derived active components play regulatory roles in mitochondrial permeability mainly through the following mechanisms: (1) Inhibiting the abnormal opening of the mPTP. The addition of the pea-seed peptide extract pea albumin 1 at 50, 100, and 200 nmol/L for 48 h effectively protected MAC-T cells from apoptosis, in part due to the suppression of ROS/NO production and the NF-κB pathway. The protein also significantly improved mitochondrial function by preventing the excessive opening of the mPTP and increasing the MMP and ATP levels [116]. Treatment with 15 µg/mL FTA for 12 h significantly decreased ROS levels in MAC-T cells and mitochondria after LPS application; reversed the changes in the protein expression of inflammatory factors TNF-α, IL-1β and IL-6; and weakened the opening of the mPTP, thereby alleviating mitochondrial swelling and damage [5]. (2) Regulating the balance of the Bax/Bcl-2 ratio. Treatment with astragaloside IV, an active component of *Astragalus membranaceus*, at 5, 10, and 20 µM for 4 h alleviated ammonia-induced mitochondrial damage and apoptosis in MAC-T cells, mainly by activating the Nrf2–ARE signalling pathway and thus inhibiting ROS production; reduced the protein levels of Bax, caspase-3 and phosphorylated p53; and significantly decreased the Bax/Bcl-2 ratio [117]. Similarly, the Bax/Bcl-2 ratio and the protein expression levels of caspase-9 and caspase-3 in the mammary glands of sheep were significantly decreased after dietary supplementation with rutin, a flavonoid compound, at 50 and 100 mg/kg body weight for 28 days, avoiding the destruction of mitochondrial permeability, preventing mitochondrial apoptosis induced by inflammation and oxidative stress, and improving mammary gland health [118]. Treatment with 50 µM quercetin and 65 µM curcumin for 30 min reduced the formation of ROS in the milk-derived neutrophils of cows with mastitis, upregulated the mRNA expression of Bcl-2 by 2.0–2.6 times, and inhibited the increase in mitochondrial permeability, an early event in apoptosis [119]. (3) Maintaining the stability of the MMP. The addition of catechins, the main polyphenolic compounds in green tea, at 5 µM for 12 h activated the Nrf2 antioxidant signalling pathway and inhibited the phosphorylation of p38 MAPK, thereby reversing the decrease in the MMP caused by H_2_O_2_ and the increase in mitochondrial permeability and preserving the normal function of BMECs; Nrf2 knockdown significantly weakened these protective effects [120]. Three hours of treatment with 10, 25, and 50 µg/mL caffeic acid, a phenolic compound widely present in the diet, effectively inhibited the LPS-induced decrease in the MMP in BMECs in a dose-dependent manner, prevented mitochondrial membrane leakage, and reduced the release of apoptotic factors [121]. Oral administration of resveratrol to mice at 30, 60, or 120 mg/kg for 10 weeks or in vitro administration to MAC-T cells at 100 µM for 24 h alleviated mitochondrial damage induced by high levels of nonesterified fatty acid stress, including restoration of the MMP, reduced mitochondrial swelling, increased ATP production, and rebalancing of NAD^+^/NADH levels. This process prevents apoptosis induced by oxidative stress and changes in mitochondrial permeability [122].

## 5. Limitations and Challenges

Although the targeted regulation of mitochondria has become a research hotspot in recent years, current research on the prevention and control of bovine mastitis with plant extracts through the regulation of mitochondrial function is still in its infancy, and direct in vivo evidence mainly reflects cell-level and mouse model results. These models have provided valuable insights for understanding mitochondrial regulatory mechanisms, particularly regarding how active components of plant extracts modulate core processes, including mitochondrial autophagy, biosynthesis, dynamics, ROS production, calcium homeostasis and permeability through specific pathways and targets, thereby protecting the mammary gland and cells from damage caused by inflammation and oxidative stress. However, a significant limitation is the lack of direct evidence for translating these mechanistic findings from model systems to dairy cows with mastitis, as these models cannot fully reflect the specific physiological processes that occur in dairy cows, such as the metabolic degradation of plant compounds in the rumen and the effective penetration of active ingredients into mammary tissue.

Fortunately, research on mitochondrial targeting mechanisms—including mitochondrial permeability, mitochondrial calcium homeostasis, and mitochondrial biogenesis— has begun to extend to ruminants. Dietary supplementation with rutin in transition sheep can inhibit the activation of the mitochondrial apoptosis pathway in the mammary glands induced by oxidative stress and inflammatory response, thereby alleviating mastitis [118]. In addition, some studies have explored the “in vivo observation—cellular mechanism—in vivo verification” pathway on the basis of clinical samples from cows with mastitis. Through transcriptome analysis, one study revealed that oxidative stress, MAM, and MFN2 are strongly involved in mastitis in the mammary tissues of cows; experiments in MAC-T cells revealed that MFN2 is a critical target for restoring MAM integrity and alleviating oxidative stress under H_2_O_2_ stimulation, thereby contributing to mitochondrial homeostasis. Furthermore, a mammary infusion of *G. lemaneiformis* polysaccharide, a natural MFN2 agonist, alleviated inflammatory responses in mastitic cows [98]. Another study similarly revealed that PGC-1α activation is impaired in the blood of cows with mastitis. A BMEC model was subsequently constructed and showed that resveratrol could activate PGC-1α through the PRKAA1 pathway, promote mitochondrial biosynthesis, and alleviate inflammatory reactions [61]. These observations have laid the groundwork for subsequent investigations into plant extracts that, following absorption and transformation in cows, act on mitochondrial functions to control mastitis. Nevertheless, the targeting and regulation of mitophagy, mitochondrial dynamics, and mitochondrial ROS in the bovine by plant extracts remain to be further validated.

Overall, multiple translational challenges still exist that need to be overcome before the active ingredients in plant extracts can be applied in clinical practice to control bovine mastitis. (1) Standardization of quality control. The active ingredients in plant extracts differ substantially in terms of the place of origin of the plant, harvesting period, and extraction process [123]. Consequently, the lack of consistent quality control criteria makes it difficult to compare findings across studies. (2) Identification of active ingredients. The separation and identification of bioactive components from plant extracts are critical steps in elucidating the chemical basis underlying their mitochondrial-targeted regulatory effects, particularly given that multiple constituents may exhibit synergistic or antagonistic interactions [124]. (3) Dose–response relationship. The modulation of mitochondrial function by active components from plant extracts is often dose-dependent [121]. Nevertheless, a single dosage level has been adopted in the majority of previous investigations, rendering it difficult to ascertain the optimal effective range of plant extracts. Accordingly, the divergent effects at different doses remain to be systematically characterized. (4) Pharmacokinetic and pharmacodynamic evaluation. Pharmacokinetics determines whether plant-derived active compounds can reach mammary mitochondria at effective concentrations to exert their pharmacodynamic actions. However, data on the absorption, distribution, metabolism, and excretion of these compounds in bovines are extremely limited. (5) Toxicity assessment. Even compounds that appear safe in cell or rodent models may not be safe in bovines. To this end, compounds should be evaluated to determine their potential impacts on rumen fermentation, hepatic metabolism, milk production, immune function, and so on. (6) In vivo efficacy tests. Finally, the clinical value of plant extracts must be validated through controlled clinical trials in bovines with experimentally induced or naturally occurring mastitis.

## 6. Conclusions

**Figure 7:**
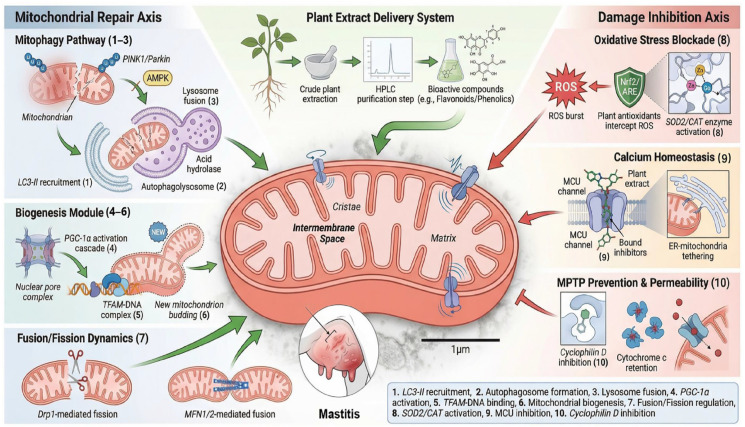
Mitochondrial-targeting mechanisms of action of plant extracts in the control of mastitis. Plant extracts can control mastitis by promoting mitochondrial repair (mitophagy, mitochondrial biogenesis, and mitochondrial dynamics) and inhibiting mitochondrial damage (mitochondrial ROS overproduction, mitochondrial Ca^2+^ imbalance, and mitochondrial permeability damage). Green arrows indicate promoting effects, and red arrows indicate inhibitory effects.

Recent studies have shown that interfering with the balance between mitochondrial repair and damage directly regulates important physiological processes, such as redox homeostasis, inflammatory processes and apoptosis, in mammary cells, providing a theoretical perspective for the control of bovine mastitis. Moreover, plant extracts, which are rich in polyphenols, alkaloids, polysaccharides, lignin, saponins and other active ingredients, have shown great potential for the targeted regulation of mitochondrial function and alleviation of mastitis and may serve as green alternatives to antibiotics (Figure 7). The active components of plant extracts may promote mitochondrial repair and inhibit mitochondrial damage by the following mechanisms: (1) regulation of mitophagy through the PINK1/Parkin or AMPK/ULK1 axis; (2) alteration of the gene and protein expression of MFN1/MFN2 and Drp1/Fis1 for maintaining mitochondrial dynamics balance; (3) promotion of mitochondrial biogenesis through the PGC-1α/NRF-1/TFAM axis; (4) scavenging of free radicals and activation of the Nrf2/ARE pathway for reducing ROS accumulation; (5) targeting of the IP3R/GRP75/VDAC1/MCU axis or MFN2 for mitigating mitochondrial calcium homeostasis imbalance; and (6) stabilization of the mPTP, the Bax/Bcl-2 ratio and the MMP to prevent disruption of mitochondrial permeability (Table 1).

Although plant extracts and their bioactive compounds represent promising candidates for further research, they still need standardized formulations, safety studies, pharmacological characterization, and controlled in vivo studies in dairy cows before these compounds can be considered reliable alternatives or adjuncts to conventional mastitis therapy.

## Figures and Tables

**Table 1:** Potential mechanisms of plant extracts against mastitis via mitochondrial targeting.

Mitochondrial Function	Treatment	Application Site	Regulation Mechanism	Outcomes	References
Mitophagy	Forsythiaside A (Mouse: gavage, 80 mg/mL for 7 d; MAC-T cells: 15 µg/mL, 12 h)	In vivo (Mouse) and in vitro (MAC-T cells)	Activates the PINK1/Parkin pathway or the AMPK/ULK1 pathway to promote mitophagy.	Ameliorates LPS-induced mastitis and inflammation.	[5,40]
	Nitidine chloride (25 µM, 2 h)	In vitro (BMECs)	Reduces the levels of the PINK1/Parkin pathway proteins to inhibit excessive mitophagy.	Relieves oxidative stress and apoptosis under hypoxic conditions.	[36]
	Schisandrin A (Mouse: intraperitoneal injection, 32 mg/kg)	In vivo (Mouse)	Activates the AMPK/ULK1 signalling pathway to induce autophagy.	Alleviates mastitis induced by LPS stimulation.	[41]
Mitochondrial dynamics balance	Procyanidin B2 (25 µM, 24 h)	In vitro (MAC-T cells)	Downregulates Fis1 and Drp1 mRNA expression while upregulating MFN1 and MFN2 mRNA expression to repair the mitochondrial dynamic disorder.	Ameliorates the inflammatory and apoptotic response induced by HS.	[59]
	Dihydromyricetin (25 µM, 12 h)	In vitro (BMECs)	Downregulates Fis1 and Drp1 mRNA expression while upregulating MFN1 and MFN2 mRNA expression to mitigate mitochondrial dynamic defects.	Attenuates the inflammatory response induced by HS.	[60]
	Resveratrol (15 µmol/L, 12 h)	In vitro (BMECs)	Upregulates MFN1 and MFN2 mRNA expression and downregulates Drp1 mRNA expression to repair mitochondrial dynamic balance.	Alleviates LPS-induced apoptosis and inflammatory responses.	[61]
	Nicotinamide mononucleotide (150 µM, 24 h)	In vitro (BMECs)	Decreases Fis1 and p-Drp1 protein expression and increases MFN1 and MFN2 protein expression to mitigate mitochondrial dynamic defects.	Improves the oxidative damage induced by HS.	[10]
Mitochondrial biogenesis	Resveratrol (15 µmol/L, 12 h/24 h)	In vitro (BMECs)	Stimulates the PGC-1α/NRF-1/TFAM signalling axis and promotes mitochondrial biogenesis.	Regulates LPS-induced inflammation and oxidative stress.	[61,70]
	Sulforaphane (10 µmol/L, 24 h)	In vitro (BMECs)	Activates the PGC-1α/NRF-1/TFAM signalling axis and triggers mitochondrial biogenesis.	Alleviates the oxidative damage induced by acetoacetate.	[69]
Mitochondrial ROS	Curcumin (10 µM, 24 h)	In vitro (MAC-T cells)	Activates the Nrf2-ARE pathway and decreases ROS levels.	Alleviates LPS-induced oxidative stress and inflammatory responses.	[86]
	Hesperidin (120 µg/mL, 24 h)	In vitro (BMECs)	Mediates the Keap1/Nrf2/ARE pathway to scavenge ROS.	Mitigates the oxidative damage induced by H_2_O_2_.	[87]
	Hydroxytyrosol (10, 25 µM, 1 h)	In vitro (MAC-T cells)	Triggers the Nrf2 signalling pathway and reduces ROS levels.	Reduces the effects of LPS stimulation.	[88]
	*Taraxacum officinale* (10, 50, 100, 200 µg/mL, 48 h)	In vitro (MAC-T cells)	Activates Nrf2/ARE pathway and inhibits ROS production.	Alleviates LPS-induced oxidative stress status.	[89]
	Lentinan (100 µg/mL, 6 h)	In vitro (BMECs)	Scavenges free radicals or activates the Nrf2/ARE pathway to reduce mtROS levels.	Reduces damage induced by LPS.	[91]
	*Astragalus* polysaccharide (Mouse: gavage, 50, 100, 200 mg/kg, 6 d; MAC-T cells: 100 µg, 24 h)	In vivo (Mouse) and in vitro (MAC-T cells)	Controls ROS/NLRP3 signalling and inflammatory factor expression.	Mitigates mastitis and restores the redox balance disrupted by LPS.	[92]
Mitochondrial calcium homeostasis imbalance	β-carotene (Mouse: gavage, 100 mg/kg, 14 d; BMECs: 80 µM, 6 h/12 h)	In vivo (Mouse) and in vitro (BMECs)	Mediates the IP3R/GRP75/VDAC1/MCU axis, inhibits the STIM1/ORAI1 axis, and maintains calcium homeostasis.	Reduces LPS-induced mastitis and inflammatory injury.	[104,105]
	*G. lemaneiformis* polysaccharides (Bovine: mammary infusion, 1 mg/mL, 10 d; MAC-T cells: 100 µg/mL,12 h)	In vivo (Bovine) and in vitro (MAC-T cells)	Suppresses pro-inflammatory cytokines, decreases SCC, and enhances milk yield. Targets MFN2 to maintain calcium homeostasis.	Alleviates mastitis and weakens inflammatory responses and oxidative stress in cells under H_2_O_2_.	[98]
	Ryanodine (10 µmol/L, 30 min)	In vitro (MAC-T cells)	Avoids Ca^2+^ overload and mtROS excess.	Prevents calcium homeostasis imbalance under LPS treatment.	[97]
Mitochondrial permeability	Pea albumin 1 (50, 100, 200 nmol/L, 48 h)	In vitro (MAC-T cells)	Prevents excessive mPTP opening, increases MMP and ATP levels, and inhibits elevations in mitochondrial permeability.	Protects cells from LPS-induced apoptosis.	[116]
	Forsythiaside A (15 µg/mL,12 h)	In vitro (MAC-T cells)	Mitigates mPTP opening and inhibits mitochondrial permeability destruction.	Reverses the inflammatory injury induced by LPS treatment.	[5]
	Astragaloside (5, 10, 20 µM, 4 h)	In vitro (MAC-T cells)	Decreases the Bax/Bcl-2 ratio and inhibits the mitochondrial permeability disruption.	Alleviates the injury and apoptosis induced by ammonia.	[117]
	Rutin (Dietary supplementation, 50 and 100 mg/kg of body weight, 28 d)	In vivo (Sheep)	Decreases the Bax/Bcl-2 ratio and inhibits increases in mitochondrial permeability in the mammary gland.	Alleviate mastitis during the transition period.	[118]
	Quercetin and curcumin (50, 65 μM, 30 min)	In vitro (milk-derived neutrophils of mastitic cows)	Upregulates the mRNA expression of Bcl-2 and inhibits increases in mitochondrial permeability and early apoptosis.	Dissipates the apoptotic response.	[119]
	Catechins (5 μM,12 h)	In vitro (BMECs)	Reverses MMP reductions and permeability alterations.	Reduces the effect of H_2_O_2_ treatment on normal functioning.	[120]
	Caffeic acid (10, 25, 50 µg/mL, 3 h)	In vitro (BMECs)	Avoids reductions in MMP, reduces the release of apoptosis factors, and inhibits increases in mitochondrial permeability.	Ameliorates LPS-induced apoptosis.	[121]
	Resveratrol (Mouse: oral administration 30, 60, 120 mg/kg, 10 weeks; MAC-T cells: 100 µM, 24 h)	In vivo (Mouse) and in vitro (MAC-T cells)	Restores the MMP, reduces mitochondrial swelling, and inhibits mitochondrial permeability impairment.	Alleviates oxidative injury induced by nonesterified fatty acid-related stress.	[122]

## Data Availability

No new data were created or analyzed in this study.
